# Dual-Stream deep learning for multimodal feature fusion and classification of balance control in elite freestyle aerial skiers

**DOI:** 10.1371/journal.pone.0337296

**Published:** 2026-07-28

**Authors:** Xinze Cui, Jie Gao, Pengquan Zhang, Jingyi Yan, Yongxia Chen, Guocai Xu, Yuqi Cheng, Xin Wang, Yanming Fu

**Affiliations:** 1 School of Physical Education, Liaoning Normal University, Dalian, China; 2 College of Exercise Health, Shenyang Sport University, Shenyang, China; 3 College of Winter Sports, Shenyang Sport University, Shenyang, China; 4 School of Physical Education and Leisure, Guangdong Ocean University, Zhanjiang, China; 5 College of Exercise Science and Health, Harbin Sport University, Harbin, China; Universita Politecnica delle Marche Facolta di Ingegneria, ITALY

## Abstract

Balance control is a key determinant of stable landing in elite freestyle aerial skiing. Rapid and precise identification of subtle differences in athletes’ balance-stability regulation is a prerequisite for targeted, evidence-based training. Conventional balance assessment typically relies on force-platform measurements of the center of pressure (COP) trajectory and subsequent time-, frequency-, and time–frequency–domain analyses. However, these indices have limited ability to capture the complex dynamics of postural control and to discriminate fine-scale differences in balance regulation among highly trained freestyle skiing aerials athletes.To address this limitation, we developed a dual-stream deep learning model that fuses time–frequency image features with COP-based statistical descriptors to classify subtle variations in balance regulation. Twenty-five elite freestyle skiing aerials athletes were recruited and performed quiet standing under two conditions: (i) bipedal stance on a stable surface with eyes open and (ii) bipedal stance on an unstable surface with eyes open. COP trajectories were recorded and their multiscale entropy computed; K-means clustering was used to stratify participants into high-, medium-, and low-stability groups. The extracted time–frequency and statistical features were then fed into the dual-stream deep learning framework for model training and validation.The proposed model achieved approximately 95% classification accuracy in distinguishing data-driven COP-based stability strata, suggesting potential utility for the sensitive assessment of balance-regulation patterns in elite freestyle skiing aerials athletes.

## Introduction

Balance control is one of the most important neuromechanical abilities for enhancing athletic performance, directly determining the precision and stability with which athletes execute complex movements [1]. In competitive sport, well-developed balance capacity is a key factor for achieving superior results. Nevertheless, in the early stages of injury prevention and the optimization of technical skills, there is still a lack of an objective assessment tool that is both easy to operate and highly sensitive, and that can rapidly and accurately distinguish inter-individual differences in balance-regulation strategies. This deficiency not only limits the refinement of sport-specific technical training but also constrains early warning of injury risk. Consequently, how to evaluate balance control conveniently and precisely has become a central research question in sport science.In skill-based disciplines that demand exceptionally high levels of coordination and control, superior balance ability has a direct impact on the accuracy and stability of movement execution [2,3]. Freestyle aerial skiing provides a representative example: athletes must maintain exquisitely fine-tuned dynamic postural control to achieve optimal performance and to avoid technical errors [4]. During the 2018 PyeongChang Winter Olympic Games, Chinese athletes competing in freestyle aerials recorded a landing-error rate as high as 28.17%, and in the 2022 Beijing Winter Olympics this figure rose to 32.56% [5]. Such high failure rates not only compromise competition outcomes but also increase injury risk and reduce the likelihood of a timely return to sport. Therefore, timely evaluation and monitoring of balance decline are essential for both performance enhancement and injury prevention [6]. In response to this need, the present study integrates multiple COP-derived feature modalities to precisely evaluate inter-athlete differences in balance-control patterns.

Methods for classifying athletes’ balance control have traditionally relied on static posturographic analysis [7], which is primarily used to evaluate the ability to maintain equilibrium during quiet standing. Such approaches are useful to a certain extent but remain limited for detecting the complex dynamics of balance control. The COP trajectory reflects the dynamic projection of the body’s center of mass onto the support surface and is widely recognized as an important objective index of postural stability [8]. Dynamic COP-based assessments, which measure COP trajectories during quiet stance, have gained increasing attention; studies show that certain extreme displacements of the COP are closely associated with future fall incidence [9].

Conventional COP analyses largely rely on global time-domain and frequency-domain metrics to describe sway magnitude and spectral energy distribution [10]. However, balance control is inherently non-stationary. Global indices cannot fully capture the localized, transient adjustments that occur during posture regulation. As a result, when balance-control capacity begins to deteriorate, single-domain time or frequency measures often lack the sensitivity to detect subtle changes [8] and cannot comprehensively reflect the moment-to-moment adaptations required to maintain equilibrium.Time–frequency analysis provides a richer dynamic description by transforming one-dimensional COP time series into two-dimensional time–frequency images, simultaneously revealing local temporal and spectral characteristics of the postural control process [11]. This approach can uncover transient increases in high-frequency components or shifts in energy distribution during balance tasks [12]. Nevertheless, time–frequency methods are not without drawbacks: they are vulnerable to signal noise, and high-frequency regions may contain artifacts unrelated to genuine postural regulation, diluting the proportion of useful low-frequency energy and interfering with analysis [13]. Moreover, these methods lack integrated global metrics and therefore struggle to provide clear quantitative criteria for stability classification. Without additional complementary information, the local features extracted by time–frequency analysis alone are insufficient for a full characterization of individual balance capacity.While single-domain time- or frequency-based descriptors are intuitive, they convey only global amplitude information and cannot elucidate specific control strategies [14,15]. Time–frequency features, although rich in dynamic detail and more sensitive for distinguishing subtle balance differences, often lack direct physiological interpretability and are difficult to translate into clinical or field applications [16]. Furthermore, traditional analytical workflows typically require offline post-processing, which delays the availability of results and limits the practicality of these methods for real-time training or clinical decision-making [17].

In recent years,with the rapid development of artificial intelligence [18,19],dual-stream and feature-fusion deep learning frameworks have developed rapidly across biomedical and sensing applications. For example, dual-stream CNN–LSTM architectures have been used for cuffless blood pressure estimation from PPG and ECG signals,demonstrating the value of parallel feature extraction and fusion in physiological prediction tasks [20]. Similarly, dual-stream convolutional neural networks have been applied to heart-sound classification for wearable devices, further supporting the effectiveness of parallel-branch modeling in biosignal analysis [21]. Beyond physiological monitoring, multimodal fusion frameworks integrating dynamic signatures and facial data have also shown strong performance in biometric authentication tasks [22]. In movement-related applications, multimodal EMG–EEG fusion strategies have improved upper-limb gesture classification by combining complementary information from different feature streams [23]. Related fusion-based deep learning approaches have also been explored in sports-injury diagnosis [24] and multimodal brain-network modeling [25].

**Fig 1:**
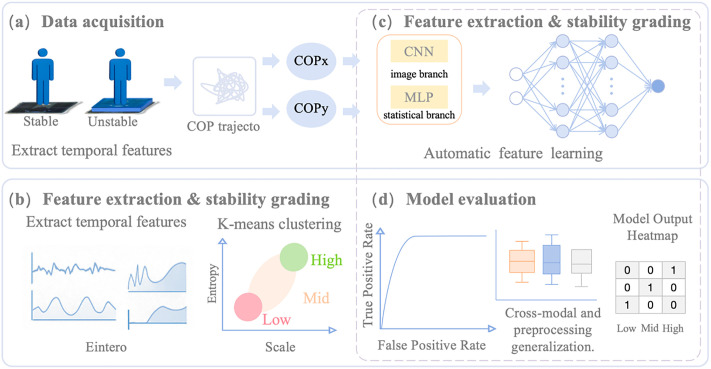
Overall workflow of the dual-stream deep learning model for balance-stability classification. (a) Data acquisition: Under stable and unstable support-surface conditions, center-of-pressure (COP) trajectories were recorded for each athlete, yielding time-series signals of COPx and COPy. (b) Feature extraction & stability grading: Multiscale complexity features of the COP signals were computed and subjected to K-means clustering, automatically stratifying participants into three stability levels: High, Mid, and Low. (c) Dual-stream feature fusion and classification: A dual-stream deep learning network was constructed in which a convolutional neural network (CNN) branch extracted time–frequency image features generated by continuous wavelet transform (CWT), while a multilayer perceptron (MLP) branch extracted statistical features. These two feature streams were fused for automated feature learning and classification. (d) Model evaluation: Ten-fold cross-validation and within-population independent validation were used to comprehensively assess the model’s generalization, class sensitivity, and robustness. Classification performance was further illustrated using receiver operating characteristic (ROC) curves, boxplots, and model-output heatmaps.

As illustrated in Fig 1,building on these insights and addressing the limitations of conventional approaches, the present study developed a dual-stream deep learning model that integrates wavelet-based time–frequency COP images with traditional statistical COP features to investigate subtle inter-individual differences in balance regulation among freestyle aerial skiers. This sport represents a highly specialized and high-risk discipline in which precise balance control is critical for landing stability, injury prevention, and performance optimization. Within this framework, a convolutional neural network (CNN) branch was used to process wavelet power-spectrum images of COP trajectories and extract time–frequency representations of postural control, while a second branch extracted time-domain and frequency-domain statistical descriptors of COP signals to characterize global stability features. The two feature streams were subsequently fused to classify balance stability levels. Because elite freestyle aerial skiers constitute a difficult-to-recruit and sport-specific population, the aim of this study was not to establish a universally generalizable model across all populations, but to examine whether a dual-stream architecture could sensitively detect subtle differences in balance regulation within this elite cohort. By doing so, this study sought to provide a practical and high-precision tool for athlete monitoring, individualized training, and early injury-risk identification.

## Materials and methods

### Participants

**Fig 2:**
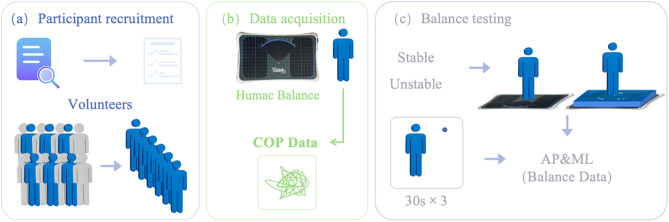
Experimental workflow of this study. (a) Participant recruitment: High-level freestyle aerial skiing athletes who met the inclusion criteria were screened and enrolled as volunteer participants. (b) Data acquisition: Each participant performed quiet-standing balance tests on both stable and unstable support surfaces. The Humac Balance platform was used to record center-of-pressure (COP) trajectory data. (c) Balance testing: For each support condition, every participant completed three 30-second trials. COP trajectories were collected in both the anterior–posterior (AP) and medio-lateral (ML) directions.

As illustrated in Fig 2,a total of 25 male freestyle aerial skiers were recruited for this study. Thirteen athletes were elite-level competitors who had participated in either the Beijing Winter Olympic Games or the World Championships (mean ± SD: age 22.50 ± 4.5 years, height 174.68 ± 2.2 cm, body mass 70.46 ± 4.2 kg). Twelve were national-level athletes who had competed in Chinese national championships (age 20.00 ± 2.8 years, height 174.87 ± 4.4 cm, body mass 69.22 ± 6.7 kg).Participant recruitment was conducted between June 1, 2022, and September 30, 2022.Before data collection, all participants provided written informed consent and completed a detailed medical history questionnaire to exclude musculoskeletal or neurological disorders that might affect balance control. None of the athletes reported lower-limb injuries or falls during the previous six months. All tests were scheduled on rest days to avoid fatigue effects. Participants were informed that they could withdraw from the study at any time without providing a reason.This study was conducted in accordance with the ethical principles of the Declaration of Helsinki and was approved by the Ethics Committee of Shenyang Sport University (Ethics Document No. [2018]9). Because the participants were elite national-team freestyle aerial skiers, including athletes who had competed in the Olympic Games or World Championships, the original individual-level data were subject to confidentiality agreements and therefore cannot be publicly disclosed.

### Data acquisition

All testing was conducted at the Key Laboratory of Winter Sports Training and Functional Evaluation of the General Administration of Sport of China. A portable balance system (Humac Balance, CSMi, USA; platform dimensions 65 cm × 40 cm) was used to record center of pressure (COP) displacements during quiet stance. The system sampled data at 100 Hz and the manufacturer’s analysis software was used to compute all COP-related parameters.To minimize environmental interference, testing was performed in a quiet, temperature-controlled room. Sessions were scheduled on rest days to ensure that all athletes were free of fatigue. Participants stood barefoot on the balance platform, with the second metatarsal heads aligned to floor markers to standardize foot placement. They were instructed to keep the trunk as still as possible and to maintain a neutral body position with arms relaxed at the sides.

Two balance conditions were assessed:

- T1 – Bipedal stance with eyes open on a stable surface
- T2 – Bipedal stance with eyes open on an unstable support surface

**Fig 3:**
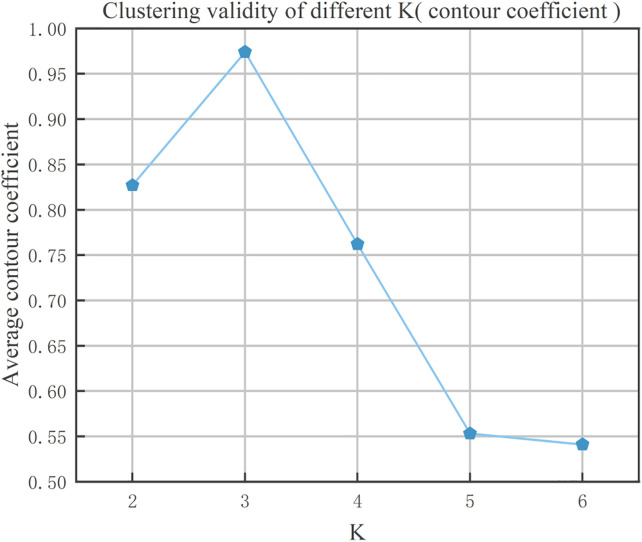
Clustering validity across different K values based on average silhouette coefficient. Multimodal Feature Extraction.

For each condition, athletes stood with their feet shoulder-width apart and maintained visual fixation on a target located 1.5 m in front of the eyes to minimize eye-movement artifacts. Each trial lasted 30 s [26]. During testing, COP excursions were continuously recorded in the anteroposterior (AP) and mediolateral (ML) directions.To improve the reliability and stability of the data, three trials were performed for each condition. Participants remained quietly seated for 30 s between trials. For subsequent feature extraction and model training, the mean of the three trials was used to ensure independence of the training set and to enhance model generalization.Throughout the protocol, participants were provided a silent testing environment and were instructed to maintain their gaze on the visual target and to remain motionless apart from natural postural sway (Fig 3).

### Data-driven approaches

**Fig 4:**
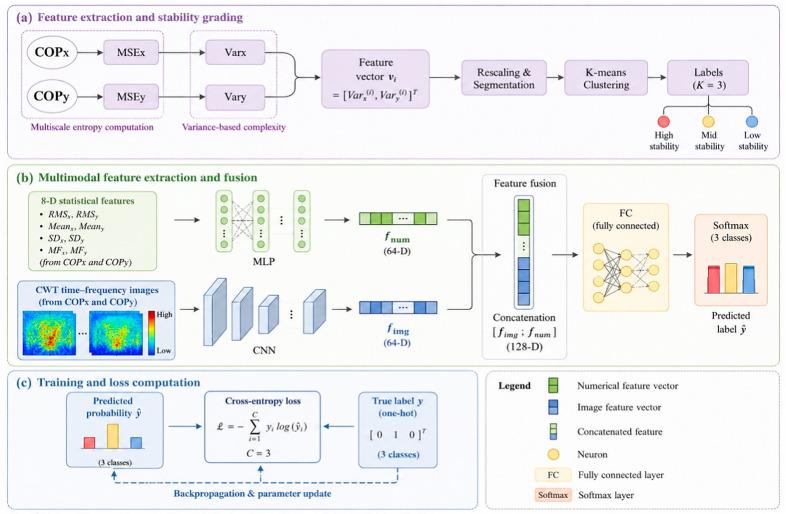
Multimodal center-of-pressure feature–fusion deep learning framework for stability classification. (a) Feature extraction and stability grading. Multiscale entropy (MSE) was computed for both COPx and COPy to obtain variance-based complexity metrics v_i. After normalization and segmentation, K-means clustering was applied to the entropy features. Silhouette analysis confirmed that K = 3 provided the optimal cluster separation, corresponding to high-, mid-, and low-stability levels. (b) Multimodal feature extraction and fusion. Numerical statistical features were processed by a multilayer perceptron (MLP), while time-frequency representations derived via continuous wavelet transform (CWT) were processed by a convolutional neural network (CNN). The learned representations from both branches were concatenated and then passed through fully connected (FC) and softmax layers for three-class stability classification. (c) Training and loss computation. The fused features were optimized using cross-entropy loss. Model outputs were visualized as class probabilities and heatmaps to illustrate classification performance on unseen samples.

As illustrated in Fig 4,the specific procedures of this study were as follows: multiscale Entropy (MSE) was first calculated from the raw center-of-pressure (COP) signals [27]. The MSE features of each participant were then entered into a K-means clustering model to automatically classify them into three stability levels—high (High), medium (Mid), and low (Low). A higher entropy value reflects stronger neuromuscular regulatory capacity and adaptability and was therefore assigned to the high-stability group, whereas a lower entropy value indicates weaker postural control and was assigned to the low-stability group, with intermediate values classified as mid-stability [28]. From the original COP signals, dual-modality features were extracted: time–frequency images generated by Continuous Wavelet Transform (CWT) to capture local dynamic characteristics of the non-stationary signal, and global statistical descriptors including the root mean square (RMS), mean, standard deviation, and mean frequency of COPx and COPy to characterize global steady-state features. A dual-stream deep learning architecture was then designed, in which the image stream based on a convolutional neural network (CNN) automatically learned time–frequency image features, while the numeric stream based on fully connected layers processed the statistical parameters; these two feature streams were integrated in a fusion layer to form a comprehensive representation of postural stability. Finally, the data were split into training and testing sets at a 7:3 ratio, and the overall performance of the model was evaluated from multiple dimensions. To further evaluate model performance on unseen samples, ten data samples from five independent participants were selected as a within-population independent validation set. These samples were labeled as high-, mid-, or low-stability groups based on the MSE clustering, and the model’s predictions were compared with the corresponding reference classifications.

To automatically identify individual postural stability labels from raw COP signals, this study adopted an unsupervised label-generation strategy based on MSE [29]. MSE shows strong sensitivity to dynamic postural changes in short non-stationary signals. By quantifying the complexity of COP signals across different time scales, it extracts key feature indices that reflect fluctuations in neuromuscular control and applies a clustering algorithm for automatic classification.Because these labels were generated in a data-driven manner from COP-derived MSE features, they were used in this study as internal stratification labels rather than as an independent external ground truth.

Each data sample contained time series of COP in the mediolateral (COPx) and anteroposterior (COPy) directions, with a uniform length of T = 3000 data points. To eliminate the influence of inter-individual signal-scale differences, each segment was first subjected to Z-score normalization:


$$\begin{array}{c} {\text{x}}_{\text{t}}^{\prime }=\frac{{\text{x}}_{\text{t}}-\mu_{\text{x}}}{\sigma_{\text{x}}},{\text{y}}_{\text{t}}^{\prime }=\frac{{\text{y}}_{\text{t}}-\mu_{\text{y}}}{\sigma_{\text{y}}},\text{ t }=\text{1},\text{T} \end{array}$$ (1)

where${\text{ x}}_{\text{t}}^{\prime },{\text{y}}_{\text{t}}^{\prime }$ are the normalized time-series values; ${\mu_{\text{x}},\sigma }_{\text{x}}$, $\mu_{\text{y}},\sigma_{\text{y}}$ are the mean and standard deviation of the COPx and COPy series, respectively; and t denotes the time index.

To introduce multi-scale temporal dynamics, the normalized series were then coarse-grained for each scale factor s∈{1,2,3,4,5}. For scale s, a coarse-grained sequence of length T/s was constructed by averaging non-overlapping segments of length s:


$$\begin{array}{c} {\text{u}}_{\text{j}}^{\left(\text{s}\right)}=\frac{\text{1}}{\text{s}}\sum_{\text{i}=(\text{j}-\text{1})\text{s}+\text{1}}^{\text{js}}{\text{x}}_{\text{i}},\text{ j }=\text{1},\left\lfloor \frac{\text{T}}{\text{s}}\right\rfloor \end{array}$$ (2)

where i is the index of the original time point and j is the index of the coarse-grained segment.

For each coarse-grained sequence, sample entropy (SampEn) was computed with embedding dimension m = 2 and tolerance r = 0.2·σ. Here s is the scale factor and s = 1–5. ${\text{A}}_{\text{s}}$, ${\text{B}}_{\text{s}}$ represent the counts of matched sequence pairs of length m + 1 and m, respectively, under scale s. Matching was defined by Chebyshev distance.

A smaller SampEn indicates a more regular and stable signal, while a larger value reflects greater complexity of postural control.


$$\begin{array}{c} \text{SampEn}(\text{m},\text{r},\text{s})=-\text{ln}(\frac{{\text{A}}_{\text{s}}}{{\text{B}}_{\text{s}}}) \end{array}$$ (3)

For each COPx and COPy channel, the multiscale entropy values were computed at five scales, yielding the sequences:


$$\begin{array}{c} {\text{MSE}}_{\text{x}}=[{\text{SampEn}}_{\text{x}}(\text{1}),...,{\text{SampEn}}_{\text{x}}\left(\text{5})\right] \end{array}$$ (4)

$$\begin{array}{c} {\text{MSE}}_{\text{y}}=[{\text{SampEn}}_{\text{y}}(\text{1}),...,{\text{SampEn}}_{\text{y}}\left(\text{5})\right] \end{array}$$ (5)

To quantify variability across scales and represent complexity, the variance of the entropy values was calculated as


$$\begin{array}{c} \text{Varx }=\frac{\text{1}}{\text{5}}\sum {\text{s }=\text{1}}^{\text{5}}{({\text{SampEn}}_{\text{x}}(\text{s})-\overset{―}{{\text{SampEn}}_{\text{x}}})}^{\text{2}} \end{array}$$ (6)

$$\begin{array}{c} \text{Vary }=\frac{\text{1}}{\text{5}}\sum {\text{s }=\text{1}}^{\text{5}}{({\text{SampEn}}_{\text{y}}(\text{s})-\overset{―}{{\text{SampEn}}_{\text{y}}})}^{\text{2}} \end{array}$$ (7)

The final feature vector for each sample was constructed as


$$\begin{array}{c} {\text{v}}_{\text{i}}=\left[\begin{array}{c} {\text{Var}}_{\text{x}}^{\left(\text{i}\right)}\\ {\text{Var}}_{\text{y}}^{\left(\text{i}\right)} \end{array}\right]\in {\text{R}}^{\text{2}} \end{array}$$ (8)

All samples were then grouped into different stability levels using the K-means algorithm in the two-dimensional feature space {vi}i = ${\text{1}}^{\text{N}}$.

Here K = 3 (representing High, Mid, and Low stability levels),

${\text{C}}_{\text{k}}$is the set of samples in cluster k, and

$\mu_{\text{k}}$is the centroid of cluster k.

To avoid sensitivity to the initial centroids, clustering was repeated 10 times (replicates = 10) and the solution with the smallest total within-cluster sum of squares was selected as the final label assignment:


$$\begin{array}{c} \underset{\{{\text{C}}_{\text{j}}\}}{\text{min}}\sum_{\text{j}-\text{1}}^{\text{k}}\sum_{{\text{v}}_{\text{i}}\in {\text{c}}_{\text{j}}}{\left\|{\text{v}}_{\text{i}}-\mu_{\text{j}}\right\|}^{\text{2}} \end{array}$$ (9)

After clustering, the three groups were assigned stability labels (High, Mid, Low) by calculating the overall mean variance of each cluster center:


$$\begin{array}{c} \mu_{\text{k}}=\mu_{\text{k}}^{\left(\text{x}\right)}+\mu_{\text{k}}^{\left(\text{y}\right)},\text{k }=\text{1},\text{2},\text{3} \end{array}$$ (10)

where $\mu_{\text{k}}^{\left(\text{x}\right)}$, $\mu_{\text{k}}^{\left(\text{y}\right)}$ are the cluster-wise averages of ${\text{Var}}_{\text{x}}$, ${\text{Var}}_{\text{y}}$, respectively.Clusters were ranked by the mean of $\mu_{\text{k}}^{\left(\text{x}\right)}$, $\mu_{\text{k}}^{\left(\text{y}\right)}$:the smallest mean was labeled high stability (0), the middle mean as mid stability (1), and the largest mean as low stability (2).The effectiveness of choosing K = 3 was validated using the silhouette coefficient (S), which evaluates clustering quality:


$$\begin{array}{c} \text{s}(\text{i})=\frac{\text{b}(\text{i})-\text{a}(\text{i})}{\text{max}\left\{\text{a}(\text{i}),\text{b}(\text{i})\right\}}\in [-\text{1},\text{1}] \end{array}$$ (11)

where a(i) is the average distance from sample i to other samples in the same cluster and b(i) is the minimum average distance from sample i to all samples in the nearest neighboring cluster.

The overall silhouette coefficient was


$$\begin{array}{c} \overset{―}{\text{s}}(\text{K})=\frac{\text{1}}{\text{N}}\sum_{\text{i}=\text{1}}^{\text{N}}\text{s}(\text{i}),\text{s}(\text{i})\in [-\text{1},\text{1}] \end{array}$$ (12)

As illustrated in Fig 3,the results showed that K = 3 yielded the highest silhouette coefficient, indicating that a three-cluster solution provided the most compact and well-separated grouping of samples and was therefore adopted as the optimal number of stability categories.

To comprehensively characterize the stability of center-of-pressure (COP) signals in the time, frequency, and time–frequency domains, numerical statistics and image-based time–frequency features were combined to construct a dual-stream input architecture.

For the numerical features, eight statistical parameters were calculated separately for the COPx and COPy directions: root mean square (RMS), mean (μ), standard deviation (σ), and mean frequency (MF, calculated using Welch’s method). These were defined as follows:


$$\begin{array}{c} \text{RMSx }=\sqrt{\frac{\text{1}}{\text{T}}\sum \text{t }={\text{1}}^{\text{T}}{\text{x}}_{\text{t}}^{\text{2}}},\text{RMSy }=\sqrt{\frac{\text{1}}{\text{T}}\sum \text{t }={\text{1}}^{\text{T}}{\text{y}}_{\text{t}}^{\text{2}}} \end{array}$$ （13）

$$\begin{array}{c} \mu_{\text{x}}=\frac{\text{1}}{\text{T}}\sum_{\text{t}-\text{1}}^{\text{T}}{\text{x}}_{\text{t}},\sigma_{\text{x}}=\sqrt{\frac{\text{1}}{\text{T}}\sum_{\text{t}-\text{1}}^{\text{T}}{({\text{x}}_{\text{t}}-\mu_{\text{x}})}^{\text{2}}} \end{array}$$ （14）

$$\begin{array}{c} \mu_{\text{y}}=\frac{\text{1}}{\text{T}}\sum_{\text{t}-\text{1}}^{\text{T}}{\text{y}}_{\text{t}},\sigma_{\text{y}}=\sqrt{\frac{\text{1}}{\text{T}}\sum_{\text{t}-\text{1}}^{\text{T}}{({\text{y}}_{\text{t}}-\mu_{\text{y}})}^{\text{2}}} \end{array}$$ （15）

$$\begin{array}{c} \text{MFx }=\frac{\sum {\text{f}}_{\text{k}}\text{Pxx}({\text{f}}_{\text{k}})}{\sum {\text{P}}_{\text{xx}}({\text{f}}_{\text{k}})},\text{MFy }=\frac{\sum {\text{f}}_{\text{k}}\text{Pyy}({\text{f}}_{\text{k}})}{\sum {\text{P}}_{\text{yy}}({\text{f}}_{\text{k}})} \end{array}$$ （16）

where $\text{Pxx}({\text{f}}_{\text{k}})$ and $\text{Pyy}({\text{f}}_{\text{k}})$ are the power spectral densities of COPx and COPy at frequency ${\text{f}}_{\text{k}}$, respectively.

The final numerical feature vector was expressed as

$\text{z }={[\text{RMSx},\mu_{\text{x}},\mu_{\text{y}},\text{MFx},\text{RMSy},\sigma_{\text{x}},\sigma_{\text{y}},\text{MFy}]}^{\text{T}}\in {\text{R}}^{\text{8}}$

At the same time, continuous wavelet transform (CWT) was applied to both COP-X and COPy signals to obtain time–frequency images.For each sample, the standardized COP-X signal x(t) and COPy signal y(t) (length T = 3000) were decomposed using a complex Morlet mother wavelet. The two directional signals were transformed separately.

Here ψ denotes the Morlet mother wavelet, s is the scale factor (300 scales evenly distributed on a logarithmic scale), and τ is the time-shift parameter. The transform yields complex coefficients on the scale–time plane:


$$\begin{array}{c} {\text{W}}_{\text{x}}(\text{s},\tau )=\int \text{x}(\text{t})\cdot \psi^{(\frac{\text{t}-\tau }{\text{s}})}\text{dt} \end{array}$$ （17）

$$\begin{array}{c} {\text{W}}_{\text{y}}(\text{s},\tau )=\int \text{y}(\text{t})\cdot \psi^{(\frac{\text{t}-\tau }{\text{s}})}\text{dt} \end{array}$$ （18）

The time–frequency magnitude maps of the two signals were then obtained as


$$\begin{array}{c} {\text{I}}_{\text{x}}(\text{s},\tau )=\left|{\text{W}}_{\text{x}}\left(\text{s},\tau \right)\right| \end{array}$$ （19）

$$\begin{array}{c} {\text{I}}_{\text{y}}(\text{s},\tau )=\left|{\text{W}}_{\text{y}}\left(\text{s},\tau \right)\right| \end{array}$$ （20）

Both magnitude maps were resampled by interpolation to a size of 64 × 64, and combined to form the dual-channel image input


$$\begin{array}{c} \text{I}\in {\text{R}}^{\text{64}\times \text{64}\times \text{2}} \end{array}$$ （21）

where channel 1 corresponds to ${\text{I}}_{\text{x}}$ and channel 2 corresponds to ${\text{I}}_{\text{y}}$.

A feature-level fusion strategy was adopted to design a dual-branch deep fusion neural network. In this model, the image features extracted by a convolutional neural network (CNN) and the statistical features extracted by a multilayer perceptron (MLP) are concatenated in a shared latent space and jointly fed into the classification layer. The two branches separately process numerical statistical features and time–frequency image features, and the feature-space concatenation enables precise modeling of the stability-classification task. The image features capture local time–frequency variation patterns of COP signals, whereas the statistical features reflect global trends and signal-energy distribution. By integrating the local spatial characteristics of time–frequency images with global statistical information, the CNN + MLP fusion model enhances classification performance [30].The network adopts a dual-stream architecture, in which image and numerical features are extracted independently by CNN and MLP before fusion.

- CNN branch: The input is a 64 × 64 time–frequency image. This branch includes two convolutional layers. The first layer uses 3 × 3 kernels with 16 filters, stride = 1, and “same” padding, followed by batch normalization and ReLU activation, and then a 2 × 2 max-pooling layer (stride = 2) for downsampling. The second convolutional layer also uses 3 × 3 kernels but increases the number of filters to 32, again with “same” padding, batch normalization, and ReLU activation. The resulting feature maps are flattened and passed through a fully connected layer to reduce dimensionality to 64, producing the image-stream feature vector.
- MLP branch: The input is a 8-dimensional handcrafted statistical feature vector. It passes through two fully connected layers, each with 64 neurons and ReLU activation, mapping the 8-dimensional input into a 64-dimensional feature space.

The 64-dimensional outputs from the CNN and MLP branches are concatenated along the feature dimension to form a 128-dimensional fused feature vector. This vector is further processed by a fully connected layer with 64 units and ReLU activation to extract higher-level fused representations, followed by the output layer and a softmax classifier that yields the three-category stability predictions.Key hyperparameters of the model include 3 × 3 convolutional kernels, a progressive increase in the number of convolutional filters (16 → 32) to capture richer features, ReLU activation for all hidden layers, and batch normalization to accelerate convergence and stabilize training. The combination of a moderate architectural scale and batch normalization effectively mitigates the risk of overfitting.

Model Input and Network Structure

The model receives two-modal input features:

(1) Time–frequency images $\text{I}\in R^{\text{64}\times \text{64}\times \text{2}}$, containing the time–frequency representations of COP-X and COPy signals;
(2) Statistical feature vector $\text{z }\in R^{\text{8}}$, including mean, standard deviation, RMS, and mean frequency.

The image branch is a convolutional neural network (CNN) used to extract local time–frequency patterns, while the numerical branch is a multilayer perceptron (MLP) used to model linear relationships among numerical features.

The two branches are fused in a joint latent space and then fully connected for stability classification.

### Forward propagation

For the CNN branch, the forward pass is defined as


$$\begin{array}{c} {\text{f}}_{\text{ing}}=\phi ({\text{W}}_{\text{2}}*\phi ({\text{W}}_{\text{1}}*\text{I}+{\text{b}}_{\text{1}})+{\text{b}}_{\text{2}})\in {\text{R}}^{\text{64}} \end{array}$$ （22）

where “*” denotes two-dimensional convolution, ϕ(·) is the ReLU activation function, and ${\text{W}}_{\text{1}},{\text{W}}_{\text{2}}$ and ${\text{b}}_{\text{1}},{\text{b}}_{\text{2}}$ are convolutional kernels and biases. After convolution, activation, pooling, and flattening, a 64-dimensional feature vector is obtained.

For the MLP branch, the forward pass is


$$\begin{array}{c} {\text{f}}_{\text{num}}=\phi ({\text{W}}_{\text{4}}*\phi ({\text{W}}_{\text{3}}*\text{I}+{\text{b}}_{\text{3}})+{\text{b}}_{\text{4}})\in {\text{R}}^{\text{8}} \end{array}$$ （23）

The fused feature for classification is then expressed as


$$\begin{array}{c} {\text{f}}_{\text{concat}}=[{\text{f}}_{\text{ing}};{\text{f}}_{\text{num}}]\in {\text{R}}^{\text{128}} \end{array}$$ （24）

To enhance the relative contribution of different feature components, a learnable feature-weighting operation was applied after feature concatenation. This operation assigns adaptive importance to each fused feature dimension during training, thereby strengthening informative representations without introducing an explicit attention module.


$$\begin{array}{c} f\text{‘}=w⊙f \end{array}$$

where,$\text{w}\in R^{\text{128}}$,learnable weight vector;⊙element-wise multiplication

Finally, the classification layer predicts the probability of three stability categories through a softmax function:


$$\begin{array}{c} \hat{\text{y}}=\text{ Softmax}({\text{W}}_{\text{5}}\cdot ({\text{W}}_{\text{6}}\cdot {\text{f}}_{\text{concat}}+{\text{b}}_{\text{6}})+{\text{b}}_{\text{5}})\in {\text{R}}^{\text{3}} \end{array}$$ （25）

where $\sum_{\text{i }=\text{1}}^{\text{3}}\hat{\text{y}}\text{i }=\text{1}$.

The deep neural network was trained using the Adam optimizer and a cross-entropy loss function.To avoid overfitting, data augmentation techniques such as random horizontal flips, rotations, and scaling were applied to increase sample diversity.Dropout regularization was used during training to randomly deactivate neurons, thus suppressing overfitting.Training was performed with a batch size of 16 for 20 epochs.

Key Adam parameters were $\beta_{\text{1}}$=0.9,$\beta_{\text{2}}$=0.999, learning rate α=1×${\text{10}}^{-\text{8}}$, and a small constant ∈ = ${\text{10}}^{-\text{8}}$ to prevent division by zero.

Parameter updates followed the standard Adam procedure:


$$\begin{array}{c} {\text{m}}_{\text{t}}=\beta_{\text{1}}{\text{m}}_{\text{t}-\text{1}}+(\text{1}-\beta_{\text{1}})\nabla_{\theta }{\text{L}}_{\text{t}} \end{array}$$ （26）

$$\begin{array}{c} {\text{v}}_{\text{t}}=\beta_{\text{2}}{\text{v}}_{\text{t}-\text{1}}+(\text{1}-\beta_{\text{2}})\nabla_{\theta }^{\text{2}}{\text{L}}_{\text{t}} \end{array}$$ （27）

$$\begin{array}{c} \hat{\text{m}}\text{t }=\frac{{\text{m}}_{\text{t}}}{\text{1}-\beta_{\text{1}}^{\text{t}}},\hat{\text{vt}}=\frac{{\text{v}}_{\text{t}}}{\text{1}-\beta_{\text{2}}^{\text{t}}} \end{array}$$ （28）

$$\begin{array}{c} \theta_{\text{t}+\text{1}}=\theta_{\text{t}}-\frac{\alpha \hat{\text{m}}\text{t}}{\sqrt{\hat{\text{v}}\text{t}}+\in } \end{array}$$ （29）

Here C = 3 is the number of categories, y is the true label, $\hat{\text{y}}\text{i}$ is the predicted probability, and the cross-entropy loss is


$$\begin{array}{c} \text{L}=-\sum_{\text{i}=\text{1}}^{\text{C}}{\text{y}}_{\text{i}}\text{log}(\hat{\text{y}}\text{i}) \end{array}$$ （30）

The detailed architecture and key hyperparameter settings of the proposed dual-stream model are summarized in Table 1.

**Table 1:** Model architecture and hyperparameters of the proposed dual-stream deep learning framework.

Component	Setting/ Description
Input 1	Time–frequency image derived from CWT of COPx and COPy
Input image size	64 × 64
Image channels	2 (COPx and COPy)
Input 2	Handcrafted statistical feature vector
Statistical feature dimension	8
Statistical features	$\text{RMSx},\mu_{\text{x}},\mu_{\text{y}},\text{MFx},\text{RMSy},\sigma_{\text{x}},\sigma_{\text{y}},\text{MFy}$
CNN branch	Two convolutional layers
Conv layer 1	16 filters, kernel size 3 × 3, stride = 1, padding = same
Activation after Conv1	ReLU
Normalization after Conv1	Batch normalization
Pooling after Conv1	Max-pooling, 2 × 2, stride = 2
Conv layer 2	32 filters, kernel size 3 × 3, stride = 1, padding = same
Activation after Conv2	ReLU
Normalization after Conv2	Batch normalization
CNN output	Flatten + fully connected layer to 64-dimensional feature vector
MLP branch	Two fully connected layers
MLP input dimension	8
Hidden units per MLP layer	64
Activation in MLP	ReLU
MLP output	64-dimensional feature vector
Fusion strategy	Feature concatenation
Fused feature dimension	128
Post-fusion layer	Fully connected layer, 64 units, ReLU
Output layer	Softmax classifier
Number of classes	3 (High, Mid, Low)
Loss function	Cross-entropy loss
Optimizer	Adam
Learning rate	1 ×${\text{10}}^{-\text{3}}$
Adam β1	0.9
Adam β2	0.999
Adam ε	1 ×${\text{10}}^{-\text{8}}$
Batch size	16
Epochs	20
Dropout	Applied during training
Data augmentation	Random horizontal flips, rotations, and scaling
Train/test split	70%/ 30%
Validation strategy	Ten-fold cross-validation + within-population independent validation

## Results

### Classification accuracy and robustness of the dual-stream model

To comprehensively evaluate the dual-stream deep learning model, we performed a multi-dimensional analysis of its overall behavior. The goal was to characterize the model’s stability during training, its consistency across experimental conditions, its sensitivity to key discriminative features, and its feasibility for practical deployment.

**Fig 5:**
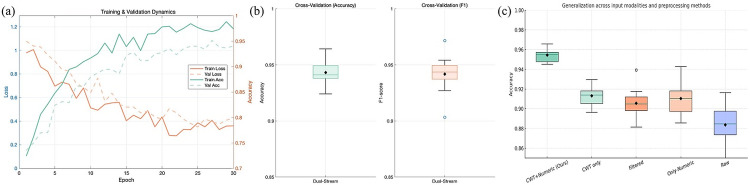
Compares the model’s performance across three key aspects: (a) Training & validation dynamics—loss (left axis) and accuracy (right axis) across epochs, with solid lines representing the training set and dashed lines representing the validation set. (b) Cross-validation performance—boxplots of accuracy and F1-score derived from ten-fold cross-validation. Boxes represent the interquartile range (IQR, 25th–75th percentile), center lines show the median, black dots the mean, whiskers 1.5 × IQR, and circles indicate outliers. (c) Cross-modal and preprocessing generalization—boxplots of accuracy under different input-modality and preprocessing settings, including CWT + Numeric (dual-stream), CWT only, Filtered, Only Numeric, and Raw conditions. Black dots denote mean values and triangles denote medians.

As illustrated in Fig 5a, training and validation losses decreased steadily as the number of epochs increased and plateaued around the 15th epoch, while accuracy converged to ≈95% on both the training and validation sets. No signs of overfitting were observed.The overall classification performance was further assessed through ten-fold cross-validation. As shown in Fig 5b, the model consistently achieved high accuracy (≈0.95) and F1-score (≈0.94) across folds. The narrow interquartile ranges and the low incidence of outliers support the internal robustness and reliability of the proposed dual-stream architecture within the current dataset..Generalization across different input modalities and preprocessing strategies is presented in Fig 5c. The dual-stream configuration (CWT + Numeric) yielded the highest mean accuracy (≈0.94) with the narrowest variation (0.945–0.965). In contrast:CWT only achieved a mean accuracy of ≈0.915 with a range of 0.895–0.925;Filtered input produced a mean of ≈0.905 (range 0.880–0.920);Only Numeric input achieved ≈0.910 accuracy with a larger fluctuation (0.885–0.935);Raw signals resulted in the lowest mean accuracy (≈0.880) and the widest variability (0.860–0.915).

### Comparative analysis with alternative deep learning models

To further benchmark the proposed dual-stream deep learning model, we systematically compared it with three state-of-the-art deep learning architectures—LSTM + Attention, Transformer + TCN, and CNN + BiLSTM—with respect to robustness, class sensitivity, and cross-condition adaptability.

**Fig 6:**
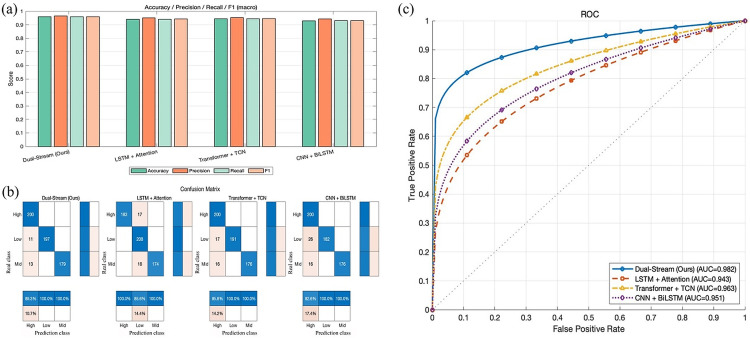
Summarizes the comparative results: (a) Macro-level performance metrics—bar charts of macro-averaged accuracy, precision, recall, and F1-score for the dual-stream model and the three comparison models. (b) Confusion matrices—three-class (High, Mid, Low) classification matrices for each model, where blue-shaded cells indicate correct predictions (with sample counts) and white cells indicate misclassifications; class-wise accuracies are reported below each matrix. (c) ROC curves and AUC values—receiver operating characteristic curves and area-under-the-curve (AUC) values for the Low-stability class treated as the positive class.

As shown in Fig 6a, the Dual-Stream model outperformed all comparators across all metrics, achieving accuracy ≈ 0.956, precision ≈ 0.959, recall ≈ 0.960, and F1-score ≈ 0.959. In contrast:LSTM + Attention reached accuracy ≈ 0.944, precision ≈ 0.946, recall ≈ 0.945, and F1-score ≈ 0.944;Transformer + TCN achieved accuracy ≈ 0.939, precision ≈ 0.940, recall ≈ 0.939, and F1-score ≈ 0.939;CNN + BiLSTM performed lowest with accuracy ≈ 0.929, precision ≈ 0.930, recall ≈ 0.929, and F1-score ≈ 0.929.The confusion matrices in Fig 6b further illustrate these differences. The dual-stream model reached 100% classification accuracy in both the Mid and Low stability groups and maintained a high recognition rate of 89.3% for the High group, with the few misclassifications concentrated within the High category. By contrast, Transformer + TCN and CNN + BiLSTM displayed markedly reduced recognition of the High-stability class (14.2% and 17.4%, respectively), while LSTM + Attention showed decreased accuracy (85.6%) for the Mid-stability group.Receiver operating characteristic analysis using the Low-stability class as the positive class (Fig 6c) confirmed the superior discriminative capacity of the dual-stream model. It yielded the largest area under the curve (AUC = 0.9822), substantially outperforming Transformer + TCN (0.963), CNN + BiLSTM (0.951), and LSTM + Attention (0.943) across the entire false-positive rate range.

### Identification of subtle differences in balance control strategies

To examine the practical applicability of the proposed dual-stream deep learning model, a within-population independent validation was performed using 10 unseen data sets that were not included in model training. Each data set was first labeled into high-, mid-, or low-stability groups through the established MSE–based K-means clustering procedure. Model predictions were then compared with these reference labels to evaluate classification accuracy and robustness on unseen samples.

Table 2 summarizes the traditional balance-test results of all participants under the two testing conditions: T1 – bipedal stance with eyes open on a stable surfaceT2 – bipedal stance with eyes open on an unstable surface.For both tasks, no statistically significant differences were detected between elite athletes and national-level athletes in the conventional COP parameters of medio-lateral displacement (COPx) and anteroposterior displacement (COPy).These findings indicate that traditional COP-based measures alone may not provide sufficient sensitivity to discriminate between elite and national-level freestyle aerial skiers, despite balance control being a critical determinant of high-level sport performance. The inability of standard metrics to capture subtle but meaningful differences further underscores the necessity and practical value of the proposed dual-stream deep learning model for precise classification of postural stability and for guiding individualized performance enhancement and injury-prevention strategies.

**Table 2:** Results of balance parameters for elite athletes and expert athletes.

Test conditions	Balance parameters	Elite athletes	Expert athletes
T1	COPx	0.04 ± 0.2	0.11 ± 0.2
	COPy	0.01 ± 0.4	−0.01 ± 0.5
T2	COPx	0.05 ± 0.9	−0.03 ± 0.7
	COPy	0.11 ± 0.4	−0.05 ± 0.5

Note: All values are expressed as mean ± standard deviation (SD).P < 0.05 indicates a significant difference between the elite and national-level athlete groups.Normality of each variable was assessed using the Kolmogorov–Smirnov test. If the data were normally distributed, independent-samples t tests were applied for pairwise comparisons; otherwise, the Mann–Whitney U test was used.

**Fig 7:**
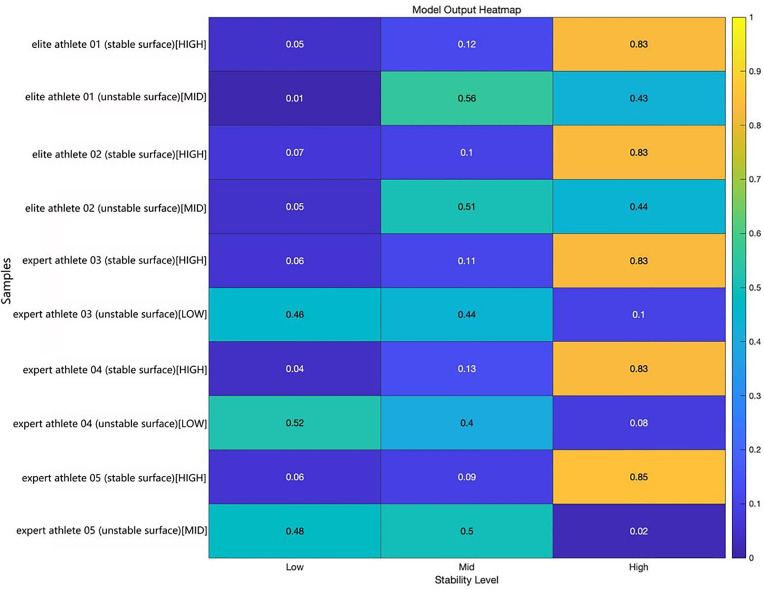
Model Output Probability Distributions Across Three Stability Levels. (a) Model output probability heatmap for individual subjects. The heatmap illustrates the predicted probabilities for the three stability levels (High, Mid, Low) across 10 external data samples (vertical axis). Color intensity, scaled by the adjacent color bar, represents the model’s predicted probability for each category.

The heatmap allows an intuitive visualization of the athlete’s current balance-stability status and its variation across testing conditions.To visualize the predictions from the within-population independent validation, Fig 7 presents the heatmaps of output probabilities for five athletes tested under two support-surface conditions, yielding 10 independent prediction instances.Under the hard-surface condition, the model’s dominant predicted class was High stability, with primary-class probabilities ranging from 0.79 to 0.93. In contrast, under the soft-surface condition, an overall downward shift in stability was observed, with predicted categories moving from High toward Mid or Low stability. Specifically, Athlete 03 and Athlete 04 were classified as Low stability, with dominant probabilities of 0.849 and 0.874, respectively. Athlete 01, Athlete 02, and Athlete 05 were predominantly classified as Mid stability, with dominant probabilities of 0.885, 0.821, and 0.817, respectively.

## Discussion

Previous research has demonstrated that COP signals are a key tool for assessing human postural stability and have been widely applied to evaluate balance-regulation mechanisms [31]. Existing evidence shows that COP sway amplitude and velocity in the anteroposterior (AP) and mediolateral (ML) directions, as well as the mean distance of the COP trajectory from its central point, are all effective indicators of postural control capacity. Smaller sway amplitudes and faster sway velocities generally indicate that an individual can return more quickly to the midline following perturbation, reflecting higher balance stability [32,33]. In addition, local dynamic measures, such as the distance between peaks in the sway-density curve, have also been shown to correlate strongly with balance ability [34]. Moreover, numerous studies have characterized COP signals using nonlinear time-series analyses, concluding that greater complexity in postural control patterns often signifies higher stability and adaptive capacity [35].

Despite these methodological advances, limitations remain in detecting subtle balance differences among elite athletes, individuals with diverse training backgrounds, or clinical populations. The principal challenge lies in the fact that traditional time-domain, frequency-domain, and time–frequency analyses emphasize global or macroscopic features, making it difficult to uncover finer-grained, latent variations in postural regulation. Consequently, there is a growing need for more refined and sensitive analytical approaches to enhance the detection of minute changes in balance ability.This need is supported by theoretical frameworks that stress the importance of improved measurement techniques to capture small but meaningful shifts in postural control. Such advances are critical for elucidating subtle inter-individual differences in balance-regulation strategies, particularly in high-performance athletic populations and specialized clinical cohorts, where precise evaluation of postural stability is essential for both performance optimization and targeted rehabilitation.

### Classification accuracy and applicability of the dual-stream model

This study used MSE, which has higher sensitivity, instead of traditional posturographic parameters to automatically identify the labels of different athletes’ balance-control stability from the raw COP signal.

Traditional posturography refers to measuring the displacement range of the COP during quiet standing, which represents the weighted average of all pressures on the surface of ground contact [36]. It is usually obtained from a platform that generates a two-dimensional time series describing COP trajectories in the AP and ML directions.Traditional measurement methods can only determine the sway distance and process over time and ignore the trajectory and range of human sway in the time series, and therefore cannot explain the temporal variations caused by complex sensorimotor integration.Simple COP fluctuations cannot reflect the periodic balance features and regularity of the human body [37]. In balance studies using COP displacement analysis, the applicability of sample-entropy methods has been demonstrated.Considering the variability, irregularity, and complexity of balance data, it is appropriate to use nonlinear tools to analyze these sequences, where entropy values in the time series characterize the degree of spatial (variability) and temporal (complexity) irregularity of movement.The limitation of sample entropy is that it does not adequately account for different time scales that may exist in a time series.In this study, multiscale entropy was applied to extend sample entropy to 15 time scales, providing additional perspectives when the relevant time scales are uncertain.This approach can effectively eliminate noise interference from external factors and measurement instruments during human balance maintenance.By calculating data and values across different scales, it can most accurately express human balance ability and determine the regularity of balance sway within periodic time series.Measurement of balance ability by MSEn can better reflect the features of athletes’ balance ability in the time series.A smaller MSEn value indicates better multisensory integration and superior balance ability [38].Conversely, a higher MSEn value suggests the need to pay more attention to potential injury risk and to formulate appropriate motor-control strategies [39]. It should also be noted that the stability labels in the present study were generated using an unsupervised MSE-based K-means procedure derived from the same COP recordings that were later used for feature extraction and model training. Although the label-generation features and model-input features were not identical, they originated from the same signal source. Therefore, the present framework should be interpreted as learning a data-driven stratification of COP-based balance-control patterns rather than predicting an independent external ground truth. From a COP-based signal-processing perspective, the combination of MSE, CWT-derived time-frequency representations, and numerical statistical descriptors provides a complementary description of postural regulation. Specifically, MSE reflects multiscale complexity, CWT captures local non-stationary dynamic features, and the statistical descriptors summarize global steady-state properties of COP behavior. Thus, the dual-stream framework is able to represent both local and global characteristics of balance regulation at the behavioral level. However, these features still remain output-level indicators derived from 30-s quiet-standing performance. They describe the observable result of postural control rather than the full neuromuscular regulation process, and therefore cannot directly reveal how sensory information is received, integrated, and transmitted into motor output during balance maintenance.

This study proposes a dual-stream deep learning model based on image features and numerical features to accurately evaluate athletes’ balance ability.In the stability-level classification task, this method partly replaces the conventional assessment approach that relied on tedious manual calculations and statistical indices.Previous studies relying only on COP trajectory statistical features showed limited ability to discriminate balance differences among high-level individuals [31,40]; single deep models easily miss dynamic changes in nonstationary signals [41,42].The dual-stream deep learning network in this study integrates the time–frequency features and numerical features of COP signals in a complementary manner.CWT (continuous wavelet transform) is used to extract dynamic features of instantaneous frequency components during balance control.Wavelet analysis can represent both time-domain and frequency-domain characteristics of signals at different time scales, thereby effectively capturing instantaneous local dynamic changes in nonstationary systems [12].Numerical features model numerical information and supplement the macro-level time-domain changes overlooked by time–frequency spectrograms.This approach overcomes the limitations of previous methods in distinguishing balance ability among high-level athletes.The multimodal feature-fusion strategy of this study is consistent with previous findings that adopted dual-stream deep models in complex nonlinear signals [20,21].For example, Venugopalan et al. proposed a multimodal architecture that fuses MRI images with clinical and genetic data in Alzheimer’s disease research and demonstrated the advantage of joint modeling of image and numerical features in disease staging [43]. Salturk et al. combined static facial images with dynamic signature trajectories, using CNN and LSTM/GRU to extract spatial and temporal features, and performed authentication classification in the fusion layer, achieving significantly higher multimodal authentication performance than any single modality [22]. Jiang et al. developed a deep learning framework for sports-injury diagnosis that fuses SPECT/CT medical images with kinematic sensor data.They proposed a convolution–Transformer hybrid model and incorporated a biomechanical-constraint module to enhance interpretability, thereby improving injury-detection accuracy [24]. Li et al. introduced a graph neural network to jointly model fMRI and EEG functional-connectivity data, realizing complementary integration of fMRI and EEG information and improving both drug-response prediction accuracy and mechanistic interpretability [44]. These studies collectively indicate that dual-stream multimodal deep fusion can significantly enhance classification and prediction performance.The dual-stream deep learning model proposed in this study follows and deepens this approach: by complementarily fusing time–frequency image features extracted by CWT with COP numerical statistical features, it achieves accurate classification of differences in balance stability among athletes.This strategy is highly consistent with previous conclusions on multimodal studies of complex nonlinear signals.The results verify the feasibility and effectiveness of the dual-stream deep learning framework based on image and numerical feature fusion for balance-control assessment.

### Differentiation of balance control strategies in athletes

Although the sample was relatively small and homogeneous, this should be interpreted in the context of freestyle aerial skiing as a highly specialized and high-risk winter sport. Elite athletes in this discipline are inherently difficult to recruit in large numbers, yet precise evaluation of balance regulation in this population remains particularly important because landing stability is directly linked to performance and injury prevention.

For freestyle aerial skiers, landing stability is of critical importance. It not only determines whether athletes can complete complex aerial maneuvers but also directly influences their competitive results and safety. In recent years, landing stability has increasingly become a decisive factor for achieving top performance in freestyle aerial skiing competitions.Wang Xin et al.[45] reported that when athletes perform high-difficulty maneuvers, the success rate typically remains at 50%–70%, whereas outstanding athletes can reach about 80%. The success rate of landing has therefore become a key factor for winning championships in freestyle aerial skiing.Although long-term balance training has improved athletes’ stability, multiple factors still influence landing balance during competition. For athletes in this discipline, landing balance is essential for achieving excellent results and ensuring personal safety.In the present study of elite and national-level athletes, we found only minor differences in balance ability between the two groups. Accurate classification using some single analytical method was therefore very challenging.In two balance tasks, no significant group differences in balance control were detected, posing higher requirements for both classification methods and data processing. Numerous previous studies have demonstrated that traditional balance features are insufficient to achieve accurate classification [46,47]. In this study, a foam pad was used to perturb balance and simulate an unstable support surface [48].The results showed that conventional analytical methods failed to effectively distinguish balance-regulation ability between elite and national-level athletes across different support conditions, whereas the dual-stream deep learning model successfully discriminated these differences.The findings further revealed that standing on an unstable surface changes neuromuscular control strategies: lower-limb muscle recruitment increases overall, antagonist co-activation is enhanced, and an increased-stiffness strategy is adopted to compensate for reduced sensory feedback and external disturbance [49]. In elite athletes, COP sway energy is mainly concentrated in low-frequency bands, indicating more refined postural control, whereas ordinary participants or those tested under unstable conditions exhibit greater high-frequency components, reflecting coarser balance regulation.These mechanisms indicate that postural adjustment under unstable support is delayed and stiffened, resulting in decreased balance ability, a pattern consistent with the dual-stream model’s detection of balance differences under surface perturbation [50].

By leveraging COP signals, the dual-stream deep learning model can accurately assess the balance-regulation ability of athletes at different competitive levels.

Previous studies have shown that high-level athletes demonstrate superior performance in balance tasks.Elite athletes can reduce the excitability of spinal and stretch reflexes through neural adaptation, thereby maintaining postural stability more effectively; in practical testing, elite shooters exhibit significantly better static bipedal balance than sub-elite athletes [1].Through multimodal fusion of COP time-domain, frequency-domain, and time–frequency features, the dual-stream deep learning model successfully captures underlying differences in neuromuscular control among athletes.The joint action of the nervous system and motor system in postural regulation can be implicitly reflected in COP motion. Previous research has demonstrated that deep learning models using COP signals can accurately evaluate the contribution of sensory inputs during balance control [50].Thus, even without EMG recordings, differences in balance strategies can still be identified, indirectly revealing variations in neuromuscular responsiveness and control strategies.Elite athletes not only possess superior balance performance but also adopt more refined coordination strategies [51].They often perform anticipatory postural adjustments to regain balance in advance [52] and during balance regulation they tend to limit or optimize redundant degrees of freedom, making movement control simpler and more efficient, while relying on precisely organized muscle synergies to maintain posture.Existing evidence shows that elite athletes exhibit a more condensed synergy structure than sub-elite athletes: while sub-elite athletes may use six muscle synergies, elite athletes require only five.In addition, major muscle groups are activated earlier and more concentrically during movement initiation in elite athletes, reflecting higher neuromuscular control efficiency and strategic advantage [53].This “fewer-but-more-effective” muscle synergy pattern indicates that elite athletes can utilize neuromuscular redundancy more efficiently, rapidly suppressing unnecessary sway.Compared with national-level athletes, elite athletes show smaller movement amplitudes and higher coordination.For example, in high-difficulty shooting tasks, elite athletes display lower amplitude and higher consistency in upper-limb and equipment trajectories, whereas ordinary athletes show greater dispersion and instability [54].These differences reflect the superior control of degrees of freedom, redundancy regulation, and synergy optimization at the neuromuscular level, consistent with the high stability and adaptability observed in elite athletes during balance tasks [51].

The dual-stream deep learning model further demonstrated its sensitivity to stability changes under different support conditions.Heat-map visualization revealed that the model can automatically focus on spatiotemporal COP features that capture subtle variations.As the COP trajectory patterns changed with surface conditions, the model’s attention regions also shifted, indicating generalization capability and robustness.Phan et al. applied a CNN to short-term COP data to predict standing-posture environments and found that CNNs maintain high accuracy even when sample size decreases or stance conditions change; CNNs can effectively distinguish environment differences even when posture changes or sampling rates decrease [55].This implies that in the present study, the dual-stream deep learning model combines time-varying frequency features captured by the CWT image branch with steady-state metrics captured by the statistical branch, creating a comprehensive representation of balance dynamics and effectively detecting stability differences induced by different support surfaces.

Within this architecture, the dual-stream fusion strategy played a pivotal role.The CWT image stream was responsible for learning time-varying postural patterns and identifying key geometric features in dynamic body processes;the COP statistical stream provided overall amplitude and distribution measures, supplementing long-term trends that the image stream might overlook.This combination of temporal and static features enabled the model to simultaneously account for instantaneous fluctuations and global trends, providing a more complete reflection of the neural mechanisms of balance control.Although the final model output is a simple class label, the dual-stream deep learning model successfully captured multimodal feature mappings, detecting deep-seated differences in control strategies among athletes.The results show that a COP-based dual-stream deep learning model can effectively distinguish balance states among athletes of different competitive levels, and that its advantage lies in representing neuromuscular control efficiency and coordination from spatiotemporal features.These findings are consistent with previous reports that deep learning–based postural recognition models not only achieve accurate classification but also have strong practical application potential [55].Therefore, the dual-stream deep learning model can be used not only for athlete training evaluation, injury prevention, and rehabilitation monitoring but also for noninvasive COP-based measurements to provide scientific feedback and guidance for athletic performance, thereby advancing the real-world application of motor-control technology.

From an application perspective, the present study focused on COP because it can be acquired more directly and efficiently in high-level athlete testing, whereas extensive acquisition of additional physiological signals such as EMG or EEG may increase measurement burden and reduce practicality in real training settings.This framework may help coaches judge the athlete’s current balance-regulation status, determine whether the difficulty of sport-specific training or landing-related tasks can be progressively increased, and monitor whether injured athletes have recovered to a satisfactory level of balance ability during rehabilitation.

## Conclusion

In the COP-based stability classification task, the dual-stream deep learning model proposed in this study demonstrated excellent performance, achieving a classification accuracy of approximately 95%.This level of accuracy satisfies the requirements for high-level athletic performance evaluation and individualized training interventions, and it effectively distinguishes subtle differences in balance control ability among elite athletes.The model also shows greater interpretability and application potential in human balance-regulation assessment.Compared with conventional black-box deep learning models, the dual-stream architecture further reveals differences in balance-control strategies between athletes of different competitive levels.Moreover, the model captures the transition from high to low stability under both hard- and soft-support conditions, intuitively reflecting adaptive adjustments in balance-control strategies, and thereby enables precise classification of balance-regulation capacity across athlete levels and environmental conditions.These features provide more accurate support for training interventions and injury-risk warning in elite athletes, and lay a solid foundation for future applications in rehabilitation medicine and sports science.

## Limitations

This study has several limitations.First, the sample was relatively small and homogeneous, consisting only of male freestyle aerial skiers from a single sport. This limits the external generalizability of the present findings. Future studies should therefore include larger and more diverse samples, including female athletes, athletes from other sports, and participants from broader competitive levels.Second, only COP data were used as input signals.Although the dual-stream architecture effectively integrates statistical and time–frequency image features, it did not incorporate additional physiological signals such as electromyography (EMG) or electroencephalography (EEG), thereby limiting insight into the deeper neuromuscular regulatory mechanisms of balance control.Another limitation is that both the class labels and model inputs were derived from COP signals, so the present framework should be interpreted as a data-driven classifier of COP-based stability patterns rather than a predictor of an independent external standard.Third, an independent unseen-sample validation was performed, the validation samples were drawn from the same athlete population and testing environment.Future research should enlarge the participant pool, incorporate multimodal physiological signals, and validate the model under conditions that better approximate real athletic environments to further enhance its universality and practical value.

## Supporting information

S1 Data Data. (ZIP)
